# Effect of Pre-Heating Prior to Low Temperature 0.1 µm-Microfiltration of Milk on Casein–Whey Protein Fractionation

**DOI:** 10.3390/foods10051090

**Published:** 2021-05-14

**Authors:** Simon Schiffer, Bello Teslim Adekunle, Andreas Matyssek, Martin Hartinger, Ulrich Kulozik

**Affiliations:** Chair of Food and Bioprocess Engineering, TUM School of Life Sciences, Technical University of Munich, 85354 Freising, Germany; Belloteslim01@gmail.com (B.T.A.); andreas.matyssek@tum.de (A.M.); martin.hartinger@tum.de (M.H.); ulrich.kulozik@tum.de (U.K.)

**Keywords:** pasteurization, serum casein, skim milk, microfiltration

## Abstract

During skim milk microfiltration (nominal pore size of 0.1 µm) at 10 °C, the whey protein purity in the permeate is reduced by an enhanced serum casein permeation, primarily of β-casein. To decrease casein permeation, the possibility of a pre-heating step under pasteurization conditions before the filtration step was investigated, so as to shift the equilibrium from soluble serum casein monomers to impermeable micellar casein. Immediately after the pre-heating step, low temperature microfiltration at 10 °C was conducted before the casein monomers could diffuse into the serum. The hypothesis was that the dissociation of β-casein into the serum as a result of a decreasing temperature takes more time than the duration of the microfiltration process. It was found that pre-heating reduced the β-casein permeation during microfiltration without significantly affecting the flux and whey protein permeation, compared with a microfiltration at 10 °C without the pre-heating step. Furthermore, the addition of calcium (5 and 10 mM) not only reduced the casein permeation and thus increased the permeate purity, defined as a high whey protein-to-casein (g L^−1^/g L^−1^) ratio, but also decreased the filtration performance, possibly due to the structural alteration of the deposited casein micelle layer, rendering the deposit more compact and more retentive. Therefore, the possible combination of the addition of calcium and pre-heating prior to microfiltration was also investigated in order to evidence the potential increase of whey protein (WP) purity in the permeate in the case of Ca^2+^ addition prior to microfiltration. This study shows that pre-heating very close to low temperature microfiltration results in an increased purity of the whey protein fraction obtained in the permeate.

## 1. Introduction

Milk protein fractionation by microfiltration (MF) is often conducted at low temperatures of around 10 °C [1,2,3]. Alternatively, it is still common to apply temperatures around 50–55 °C in order to obtain higher flux levels as a result of the decreased permeate viscosity [4,5]. Apart from reasons related to microbial growth, a point of difference between these temperatures is that the purity of the whey protein fraction in the permeate, as well as the casein yield in the retentate, are reduced at a low temperature [6] because of the enhanced migration of soluble casein, primarily β-casein, from the casein micelle into the serum [7]. To prevent the undesired migration of casein into the serum phase, a possible method could be the modification of the chemical composition of skim milk [8]. Lowering the pH of milk (pH 6.7–3.5) and/or adding calcium ions (0–30 mM) enhanced the micellar structural stability, thus decreasing the level of free caseins in the serum compared with that of unmodified milk [9,10,11]. However, manipulation of the chemical composition of skim milk prior to microfiltration can negatively affect the filtration performance because of the enhanced interactions between the casein micelles deposited in a more compact fouling layer at the membrane surface [12]. As shown by Schiffer et al. [13], the addition of calcium (0.25–30 mM) results in a reduction of casein permeation during 0.1 µm-microfiltration at 10–14 °C. However, when the 5–10 mM of added calcium was exceeded, the whey protein permeation also decreased because of the additional retention effect of a dense fouling layer of casein micelles. While adjusting the pH (6.8) of skim milk at 10 °C with added calcium (5–20 mM), there was no observable effect on the retention of β-Lactoglobulin (β-Lg) compared with filtration with added calcium without pH adjustment. Thus, the casein deposit onto the membrane surface would not be affected by such changes. Therefore, processing steps should be considered so as to counterbalance the deposit compaction, caused by the addition of calcium concentrations as high as 10 mM. Furthermore, alternative means should be investigated to decrease the casein permeation during low temperature milk microfiltration, without affecting the mass flow of the target whey proteins into the permeate. As reported by Zulewska et al. [14], a comparison of the filtration performance of milk with different thermal pre-treatments, namely thermization (65 °C, 20 s) and pasteurization (72 °C, 15 s; beforehand but not immediately before the filtration), showed only small differences regarding flux and protein permeation. As shown by Liu et al. [15] by ultracentrifugal separation, the kinetics to reach the equilibrium state between serum casein and micellar-bound casein are more rapid when heating milk in a short period of time compared with cooling in a temperature range of 10–40 °C, because the equilibration is diffusion-driven, which is faster at higher temperatures. Ali et al. [16] followed a similar approach in the context of different thermal pre-treatments of milk prior to cheese-making, in a temperature range from 30–60 °C, with a starting temperature of 4 °C. Upon cooling the milk from 60–40 °C to 10–4 °C, the time required to reach the equilibrium was found to be between 60 min and several days [15,16]. The primary structure of β-casein exhibits high amounts of nonpolar amino acids in the C-terminus [17], resulting in primarily hydrophobic interactions of this casein species with other caseins monomers structuring the micelles. During the heating process, hydrophobic interactions are strengthened and the equilibrium of β-casein is shifted toward a micellar-bound structure. Therefore, the approach in this study was to apply a pre-heating step by tempering the milk to 78 °C for a short period, the same as that for pasteurization, promptly followed by low-temperature microfiltration. The time allowing equilibrium regarding casein diffusion at a low temperature is sufficiently high to perform microfiltration immediately after this dedicated heat treatment.

Apart from binding casein monomers to the casein micelle by hydrophobic interactions induced by elevated temperatures of up to 78 °C, the addition of calcium is known to shift the equilibrium towards lower serum casein concentrations, compared with the filtration at 10 °C without added calcium, by means of ionic interaction. However, the addition of higher amounts of calcium, which are required to decrease the casein permeation to a very low level, leads to an altered structure of the deposited layer, reducing the flux and whey protein permeation. The reduced filtration performance is assumed to occur if a certain amount of free calcium is exceeded, thus increasing the interactions in the deposited casein structure on the membrane surface [13,18].

Therefore, the purpose of this study was to assess whether the combination of short-term pre-heating of milk prior to low-temperature microfiltration with or without the addition of calcium (5 and 10 mM) would result in an improved protein fractionation. Following pre-heating with or without calcium addition, the subsequent cooling should not immediately result in an increase of β-casein in the serum, which is associated with the micelle structure via hydrophobic interactions in the pre-heating step, because of the delay in casein micelle/serum casein re-equilibration towards the state at a low temperature. Despite cooling within the microfiltration unit that immediately followed the pre-heating step, β-casein release from the micelle to the serum may be limited, given that the equilibrium time allowing casein diffusion between both phases is longer than the process. Hence, an increased separation efficiency of casein and whey protein during low-temperature milk microfiltration should be achievable, provided the time between pre-heating or pasteurization and microfiltration is minimized.

Regarding the combined effects of pre-heating and with the addition of up to 10 mM of calcium, the expected result is that pre-heating also reduces the impact of surplus calcium by shifting the ion equilibrium towards the formation of casein and/or inorganic calcium complexes, promoting the formation of salt bridges between casein monomers, thus stabilizing the micellar structure prior to the filtration [19]. This is to be expected, because of the increased concentration of soluble calcium at lower temperatures [20]. Thus, deposit compaction at higher amounts of added calcium could be avoided, and flux and whey protein permeation could be kept at a high level.

## 2. Materials and Methods

### 2.1. Skim Milk and Calcium Supplemented Skim Milk

Pasteurized skim milk (74 °C for 28 s) was obtained from a local dairy company (Molkerei Weihenstephan GmbH and Co. KG, Freising, Germany) and stored up to 4 d at 4 °C until use. Although for industrial applications raw milk would be preferable to avoid two consecutive heating steps during the milk protein fractionation process, pasteurized skim milk was used to prevent enhanced growth of microorganisms or enzymatic activity. The protein distribution of the skim milk used was analyzed within this study according to Chapter 2.4, and is summarized in Table 1. Calcium enrichment was done by adding 1 M calcium chloride solution at amounts specified in the results section to skim milk prior to heat treatment, and then stirring overnight at 4 °C; the equilibration time is in accordance with Udabage et al. [11]. The pH decreased from 6.76 at native conditions to 6.62 and 6.48 after the enrichment to 5 and 10 mM calcium, respectively (measured at 10 °C with a pH-meter from Knick Elektronische Messgeräte GmbH and Co. KG, Berlin, Germany).

### 2.2. Pre-Heating before Filtration

**Figure 1:**
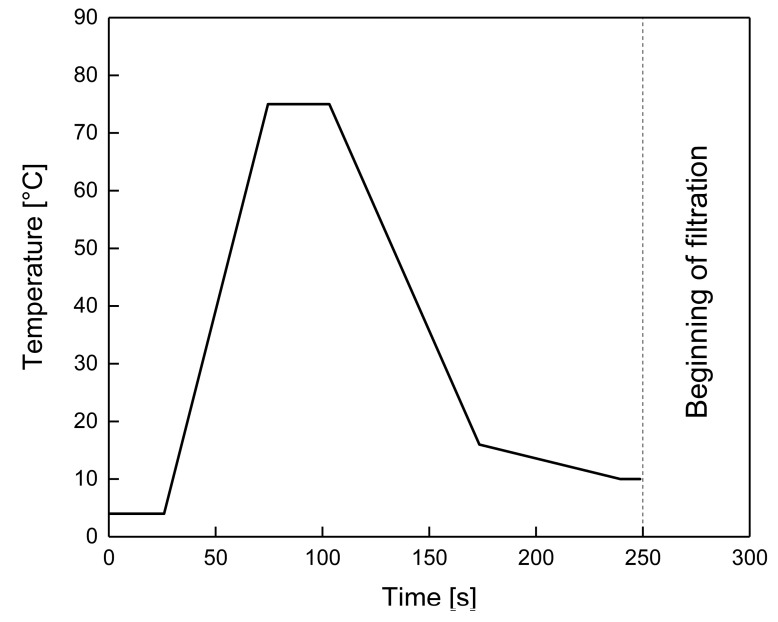
Temperature–time profile for the pre-heating step prior to the filtration.

Skim milk was pre-heated at 78 °C for 30 s in a continuous flow-through indirect tubular heat exchanger (GEA TDS GmbH, Ahaus, Germany). A volume flow rate of 80 L h^−1^ was applied in order to avoid a high amount of milk to be drained after the experiment. The heat exchanger was directly linked with the microfiltration feed tank, with a cooling section in-between to temper the milk to 10 °C (Figure 1).

### 2.3. Filtration Experiments

Three polyethersulphone (PES) membrane cassettes with a nominal pore size of 0.1 µm and an active membrane surface area of 0.093 m^2^ each (Pall, Port Washington, WI, USA) were used. Before filtration, the membranes were conditioned with 0.5% of the caustic cleaning agent Microl Mix MAT Flüssig Plus (Wigol, Worms, Germany) for 20 min at 55 °C, and afterwards, were flushed twice with softened water. Using a double-jacketed feed tank (*v* = 5 L), the temperature was kept at 10 °C. A VGS430 gear pump (Verder Deutschland GmbH and Co. KG, Haan, Germany) generated a constant volume flow of 80 L h^−1^, and a transmembrane pressure of Δp_TM_ = 1.1 bar (p_1_ = 1.2 bar at the membrane inlet, p_2_ = 1.0 bar at the membrane outlet) was adjusted. Microfiltration experiments were performed in a flow-through mode similar to that of industrial operation, so as to minimize the milk residence time in the MF unit. Both the permeate and retentate were not recirculated and instead were continuously replaced by milk directly from the pre-heating step. Without pre-heating, the skim milk was tempered at 10 °C for 60 min before the filtration experiment.

The volume flow rate was chosen, such that the milk use was low (160 L) and the feed tank filling level could thus be kept constant at a low height in order to keep the time required in the temperature range of 10 °C as short as possible. The chosen volume flow rate yielded a nominal crossflow velocity above the membrane of 0.13 m s^−1^, which is lower than in many industrial membrane units, but still at the low end of the range of conditions typical for spiral-wound polymeric membranes. Experiments without pre-heating were conducted as a control. Permeate mass flow (${\dot{m}}_{Permeate}$) was measured gravimetrically (permeate flow per min (g min^−1^)) and used to calculate the flux (*J* (L m^−2^ h^−1^)) with the active membrane area (*A_Membrane_* (m^2^)), as well as the permeate density ($\rho_{Permeate}$) at 10 °C of 1.023 kg m^−3^ (measured by a DMA 4100M density meter from Anton Paar, Graz, Austria; Equation (1)).

(1) $$J=\frac{{\dot{m}}_{Permeate}}{\rho_{Permeate}\times A_{Membrane}}$$

After reaching stable filtration conditions at 50 min, the mass flow was measured every 5 min for another 50 min. Retentate samples were taken at the beginning and at the end of the filtration, and permeate samples were taken every 10 min after the quasi-stationary phase was reached. As for the flux, the protein concentration in the permeate was measured under stable filtration conditions. After reaching stable flux conditions, no further time dependent shift in flux and protein permeation occurred at a filtration temperature of 10 °C, as also shown by Hartinger et al. [6]. Whey protein and casein permeation were calculated according to Equation (2), with the respective concentration values in the retentate at the start and end of the filtration. The protein permeation data (P) shown in Chapter 3.1 are the mean values obtained from the steady state phase of filtration (5 samples between 60–100 min filtration time).

(2) $$P=\frac{C_{Permeate}}{C_{Retentate}}\times 100\%$$

The mass flow ($\dot{m}$) was calculated according to Equation (3), using the permeate protein concentration *c_Prmeate_* and the flux (*J*).

(3) $$\dot{m}=c_{Permeat}\times J$$

At the end of filtration, the plant was flushed twice with softened water and cleaned as described by Schiffer and Kulozik [21].

### 2.4. Analyses

The concentrations of the single casein fractions κ-, α_S1_-, α_S2_-, and β-casein and the total whey protein concentrations (native and denatured whey protein) in the retentate and permeate samples were quantified by a 1:5 dilution of samples in a guanidine buffer (pH 7.2), resulting in a final guanidine concentration of 5.1 M. The quantification was performed by reversed phase high performance liquid chromatography (RP-HPLC) using a Agilent 1100 Series chromatograph (Agilent Technologies, Waldbronn, Germany) with a C18 analytical silica-based column (Agilent Zorbax 300SB-C18, 4.6 × 150 mm, 5 μm), as described by Dumpler et al. [22]. Solvent A (0.1% trifluoroacetic acid (TFA) in 90% HPLC grade water and 10% acetonitrile) and solvent B (0.07% TFA in 10% HPLC grade water and 90% acetonitrile) were used with a flow rate of 1.2 mL min^−1^ at 40 °C. Peak identification and calibration were conducted with analytical standards of the casein species and whey proteins from Sigma-Aldrich, Steinheim, Germany. The total whey protein concentration was calculated as the sum of α-lactalbumin, β-lactoglobulin A and B, bovine serum albumin, and lactoferrin. The reported total casein concentration was obtained as the sum of the single casein fractions.

To obtain the whey protein denaturation degree, the pH was adjusted to pH 4.6 with 0.01 and 0.1 M HCl, in order to precipitate the denatured whey proteins (which are insoluble at pH 4.6), and was separated by a 0.45 µm syringe filter [23]. According to Equation (4), the separated native whey proteins and the total whey proteins in the samples were compared, and thus the denaturation degree was calculated.

(4) $$Denaturation\;degree=\left(1-\frac{C_{native}}{C_{total}}\right)\times 100\%$$

### 2.5. Data Evaluation and Statistics

Data plotting and statistical data evaluation were performed using OriginPro 2017G (OriginLab Corporation, Northampton, MA, USA). Error bars represent the max/min values of two independent experiments. All of the experiments were performed as two independent replications. Statistical significances were evaluated using one-way analysis of variance (ANOVA). The calculated *p*-values are given in the text. A significance level of *p* ≤ 0.05 was defined as the minimum. 

## 3. Results

### 3.1. Effect of Pre-Heating on the Filtration Performance

The assessment criteria for the impact of pre-heating on the microfiltration performance were the flux, casein, and whey protein concentrations in the permeate. In the retentate, the casein and whey protein concentration were 33.7 g L^−1^ and 5.6 g L^−1^, respectively. As shown in Table 2, the flux remained unaffected (*p* > 0.05) and the whey protein concentration was also only slightly reduced by a pre-heating of the feed (*p* > 0.05).

In contrast with the flux and whey protein concentration, a distinct reduction of the casein concentration in the permeate was observed with the pre-heated milk, compared with filtration without pre-heating. The casein concentration decreased by approximately 35%. The β-casein concentration showed the most pronounced reduction in the permeate, from 0.32 g L^−1^ without pre-heating to 0.19 g L^−1^ with pre-heating, respectively (*p* < 0.05). However, the effect of a pre-heating only slightly reduced the concentrations of the casein species α_S1_-, α_S2_-, and κ-casein in the permeate. The reduced casein permeation during microfiltration following a pre-heating step was assumed to result from an aggregation or re-integration of formerly soluble serum caseins (primarily of β-casein). This observation is consistent with the data shown by Ali et al. [16].

Furthermore, because similar flux values were obtained without and with pre-heating, it can be concluded that no significant alteration of the deposited layer structure occurred and, thus, a possible interaction of denatured whey protein with casein micelles, which can deposit on the membrane surface, did not affect the deposit formation. However, the slight decrease in the whey protein permeation could have resulted from an increased denaturation degree from 4.7% without, to 10.2% when pre-heating was performed, respectively; thus, for an interaction of denatured whey protein with κ-casein, both soluble (i.e., in serum) and micellar (insoluble) aggregates were produced, given the covalent interaction between denatured WP (predominantly β-Lg) with κ-casein. The relative amount of each aggregate type is affected by the pH of milk as described by Anema and Li [24]. The result of this interaction is the formation of aggregates unable to pass through the membrane.

### 3.2. Effect of Pre-Heating and Calcium Addition on the Microfiltration Performance

As reported by Schiffer et al. [13], for a skim milk with microfiltration at 10 °C without pre-heating, low amounts of added calcium of up to 1 mM helped to keep mainly the β-casein permeation low; while not affecting the flux and whey protein permeation, whereas higher amounts of added calcium further reduced the casein permeation, but resulted in reduced whey protein permeation and lower flux. Here, we studied the combined effects of pre-heating and calcium addition at higher levels to assess whether a temperature induced complexation of calcium in micellar or inorganic complexes by pre-heating prior to the low-temperature milk microfiltration could be conducted. It was assumed that the impact of calcium added at levels higher than 5–10 mM on the deposit layer compaction could be decreased, and the casein permeation could also further be reduced.

**Figure 2:**
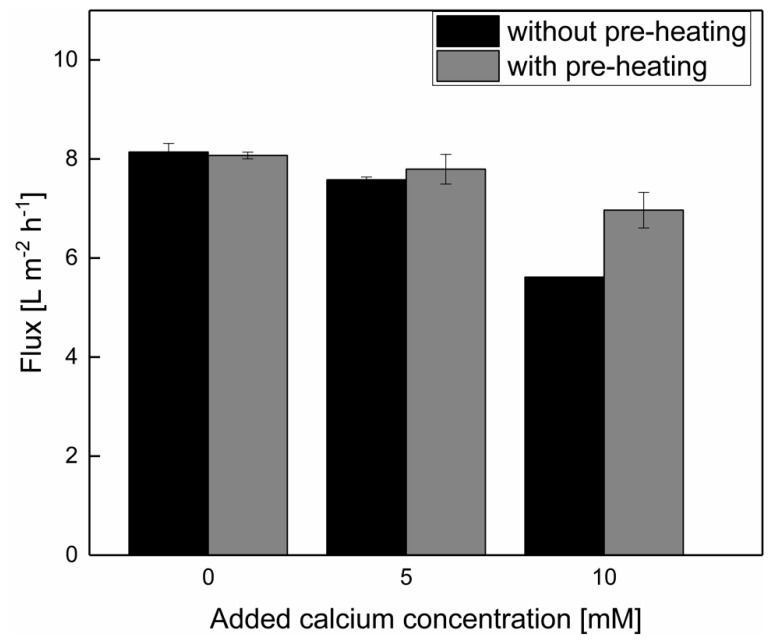
Flux during MF of skim milk with 0, 5, and 10 mM added calcium. No significant difference between the flux with and without the pre-heating of skim milk was determined for 0 and 5 mM added calcium (*p* > 0.05). A significant difference at a level of *p* < 0.05 between the flux with and without pre-heating for skim milk with 10 mM added calcium was determined.

An addition of up to 5 mM calcium did not result in a flux reduction compared with the filtration without calcium addition, either with or without pre-heating (Figure 2). However, a further increase of the calcium enrichment in skim milk of up to 10 mM calcium reduced the flux in both cases, from 8.1 ± 0.2 L m^−2^ h^−1^ without added calcium, to 5.6 ± 0.0 L m^−2^ h^−1^ without pre-heating and 7.0 ± 0.4 L m^−2^ h^−1^ with pre-heating. These findings are in accordance with results of Schiffer et al. [13] during low-temperature skim milk microfiltration. It was described that a flux reduction occurred upon the addition of 10 mM calcium because of an alteration in the deposited layer structure on the membrane surface, thus increasing the fouling resistance by a factor of 1.2.

However, as shown in Figure 2, after the addition of 10 mM calcium, the flux was reduced to different levels in the case of pre-heated milk. Without pre-heating, the flux decreased more sharply, approximately 20% below the flux of the filtration with pre-heating (*p* < 0.05). Changes in the structure of the deposit layer can obviously be reduced by pre-heating prior to microfiltration, despite the addition of 10 mM of calcium. It was shown in our previous study [13] that such a level of calcium added to milk without pre-heating led to a drastic reduction in the microfiltration performance. This can be attributed to a reduced concentration of soluble calcium in the aqueous phase by the formation of calcium complexes, as the solubility of calcium decreases with increasing temperatures [25,26,27]. Thus, as the temperature is increased, more calcium is integrated within the (insoluble) micellar phase or in inorganic complexes, and the amount of soluble calcium, which can induce salt bridges in the deposit layer during the filtration, is assumed to be reduced after pre-heating compared with the control without pre-heating, where the added calcium remains in a soluble form. It can thus induce salt bridges between individual serum caseins and between individual casein micelles in the deposited layer at the membrane surface. To conclude, up to 5 mM of calcium can be added without negative effects on the microfiltration flux, irrespective of whether there it is done with or without pre-heating step. Furthermore, the flux reduction was less pronounced upon the addition of 10 mM of calcium in the case of pre-heated milk. Therefore, upon calcium addition, a higher permeate mass flow was obtained with pre-heating compared with filtration without pre-heating.

**Figure 3:**
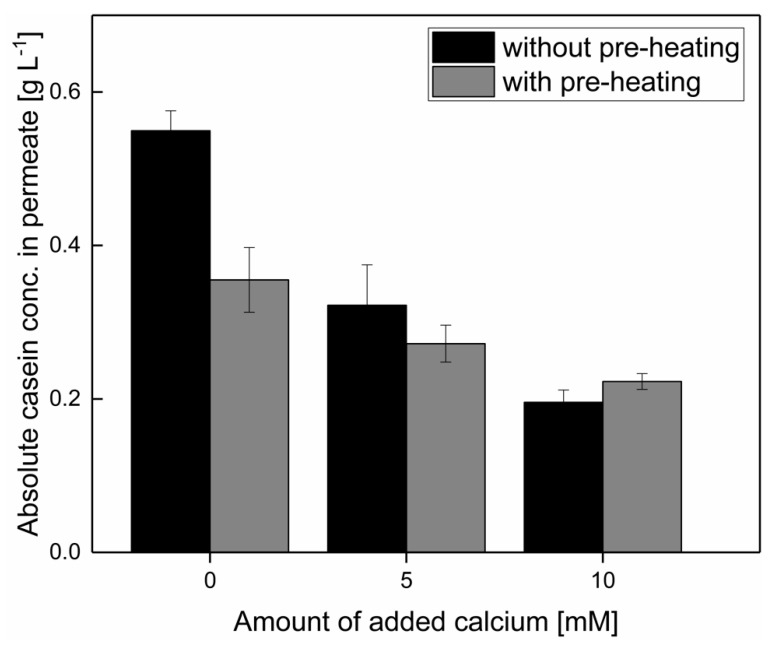
Total casein concentration in the MF permeate after the addition of 0, 5, and 10 mM of calcium.

As shown in Figure 3, the addition of 5 mM of calcium reduced the casein concentration in the permeate during microfiltration from 0.55 ± 0.03 g L^−1^ to 0.32 ± 0.05 g L^−1^ for unheated milk without and with added calcium, respectively (*p* > 0.05). This is in accordance with the results reported by Schiffer et al. [13]. The serum casein concentration decreased after the addition of 5 mM of calcium because of changes in the protein–protein interactions [9,11]. The addition of 5 mM of calcium without pre-heating reduced the casein concentration in the microfiltration permeate to the same level of approximately 0.3 g L^−1^, as in the permeate of a pre-heated and microfiltrated milk with no added calcium. Therefore, a reduction in the casein permeation can be achieved either by a calcium added induced re-integration of serum casein in the micelle or by hydrophobic interactions induced during pre-heating immediately before microfiltration at low temperatures.

However, a further decrease in casein permeation through a combination of calcium addition prior to pre-heating and filtration is only possible when up to 10 mM of calcium is added (Figure 3), because of the enhanced deposit formation after the addition of 10 mM of calcium, as described by Schiffer et al. [13]. After the addition of 5–10 mM calcium, no difference in the permeate casein concentration could be observed with or without pre-heating (*p* > 0.05). It is assumed that after the addition of 5–10 mM of calcium, the serum casein concentration was decreased because of the formation of salt bridges or from electrostatic effects. Thus, the lower level of casein in the serum after a pre-heating was obscured by the serum casein reduction resulting from the addition of calcium. The similar casein permeation in the experiments with and without thermal pre-treatment was assumed to result from the fact that serum casein can be bound by salt bridges after the addition of calcium, and thus the amount of serum casein is too low to be affected by pre-heating. It seems that not all serum casein can be integrated within casein micelles or casein aggregates, and that a certain amount of permeable casein monomers always remain. To separate the effects of calcium addition and pre-heating on the deposit layer, as well as the casein equilibrium between the micellar state and serum, a further study is suggested in order to determine the composition in the micellar phase according to the difference of content in the serum with an ultracentrifugal approach, independent of filtration.

**Figure 4:**
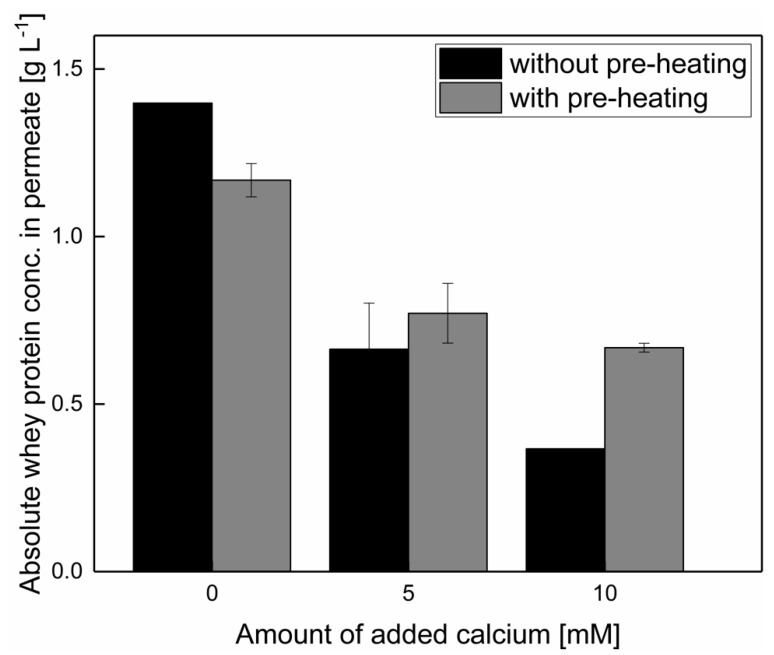
Whey protein concentration in the MF permeate after the addition of 0, 5, and 10 mM calcium.

As discussed above, the addition of calcium induced the formation of a more compact deposit, which impacted the whey protein permeation. Therefore, the effect of the calcium addition on the whey protein passage into the permeate was investigated. As can be seen in Figure 4, a calcium addition of 5 mM decreased the whey protein concentration, regardless of thermal pre-treatment before filtration. However, a further increase in the calcium addition to 10 mM resulted in a lower impact on the reduction of whey protein permeation when pre-heating was conducted. The denaturation degree of whey protein was slightly enhanced after a pre-heating of calcium enriched skim milk from 10.2 ± 0.8% without added calcium, to 11.4 ± 0.8 and 12.6 ± 0.0% with 5 and 10 mM added calcium, respectively. Without pre-heating, the denaturation degree remained at 4.7 ± 1.7%, independent of calcium addition. In this case, the lower impact of the addition of 10 mM calcium was in accordance with the effect on flux (Figure 2). The enhanced whey protein permeation with 10 mM of added calcium with pre-heating was assumed to occur because of the calcium complexation by the micelle, i.e., reduced activity of the ionic calcium, compared with a filtration with the same amount of added calcium, but without pre-heating.

However, a certain structural effect in the deposited layer upon the addition of 5–10 mM of calcium must have occurred, which could not be counterbalanced by pre-heating and related complexation of calcium. Even though pre-heating can lower the impact on whey protein permeation induced by the addition of 10 mM of calcium, the addition of 5 or 10 mM of calcium reduced the whey protein permeation in any case.

### 3.3. Impact of Pre-Heating on Casein and Whey Protein Mass Flow through the Membrane

**Figure 5:**
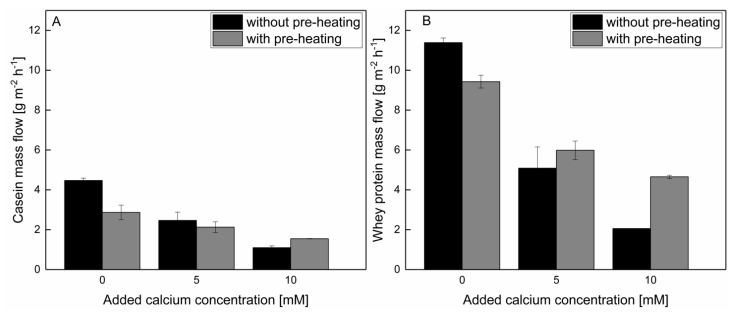
(**A**) Casein and (**B**) whey protein mass flow of a filtration of skim milk with 0–10 mM added calcium, with and without prior pre-heating.

In the case of milk protein fractionation, the protein mass flow of casein and whey protein in combination with the whey protein/casein ratio (WP/C ratio) can be used to compare the filtration performance as a decisive criterion. As can be seen in Figure 5A, the casein mass flow can either be reduced by approximately 36% by pre-heating prior to the microfiltration, or by approximately 45–52% through the addition of 5 mM calcium compared to a low-temperature filtration without pre-heating and calcium addition. A further reduction of the casein mass flow in permeate is possible if 10 mM of calcium is added. Concluding, regarding casein mass flow, either pre-heating or the addition of calcium can be used. A combination of pre-heating and calcium enrichment does not reduce the casein mass flow compared with filtration without pre-heating with the same level of added calcium (Figure 5A). However, a high whey protein mass flow is only obtainable if no calcium is added (Figure 5B). The addition of 5 and 10 mM of calcium reduced the whey protein mass flow compared with filtration without both calcium addition and pre-heating procedures by approximately 47–55%, and therefore, counterbalanced the positive effect of a reduced casein mass flow. After the addition of 10 mM of calcium, pre-heating can increase the whey protein mass flow compared with filtration without thermal pre-treatment, but at a lower extent compared with filtration without both the addition of calcium and pre-heating procedures. Therefore, an addition of 5–10 mM of calcium with and without pre-heating is detrimental to obtain a high whey protein mass flow, even though pre-heating can reduce the negative effect of calcium addition on the filtration performance.

An increasing WP/C ratio in the permeate, and therefore a higher purity, can be obtained if pre-heating is conducted prior to microfiltration compared with filtration without pre-heating (Table 3). If calcium is added to skim milk without pre-heating, the WP/C ratio is even reduced, due to an enhanced formation of the deposit layer, whereas pre-heating can partly counterbalance this effect. Even though the same purity (WP/C ratio) with a filtration processed using a pre-heating step with a calcium enrichment of 0, 5, and 10 mM can be obtained, the reduced whey protein mass flow eliminates this positive effect, reducing the filtration efficiency. Therefore, a pre-heating step can be used to increase the filtration performance, whereas the addition of 5–10 mM of calcium does not have an additional positive effect. 

## 4. Conclusions

Pre-heating prior to the microfiltration of skim milk at 10 °C can be applied to reduce casein permeation during milk protein fractionation. Because of the longer equilibration times between soluble and micellar caseins upon decreasing temperatures, the low serum casein concentrations in the un-equilibrated feed can be utilized to reduce the casein permeation, if microfiltration at low temperatures is performed directly after a pre-heating step. The pasteurization of skim milk, which is a mandatory step in milk processing, could be such a pre-heating step. This means that a combination of pasteurization shortly after microfiltration for milk protein fractionation can improve the purity of the whey protein fraction in the permeate, without additional processing steps. Pre-heating can be applied to mainly reduce the level of soluble β-casein and its presence in the permeate. This processing option combines the positive effects of low serum casein concentrations (which otherwise can only be achieved at high filtration temperatures) and low microbiological growth and activity. Thus, with the process presented in this study, a combination of processes already existing in the industry can be used to optimize microfiltration at low temperatures. Compared with filtration at high temperatures, pre-heating prior to cold milk microfiltration could alternatively be applied to achieve a high permeate purity in terms of the casein/whey protein ratio. However, the reduced flux, as a consequence of the higher viscosity at low temperatures, in any case leads to a reduced whey protein mass flow.

As an alternative to applying a pre-heating step, the addition of calcium also reduces the amount of casein in the permeate; however, at the cost of a lower filtration performance with regard to flux and whey protein permeation. This study demonstrates that a combination both pre-heating and the addition of calcium can enhance the flux and whey protein permeation. It would be of interest to apply the insights from this study in scale-up experiments performed under conditions close to industrial situations. In particular, it should be noted that the crossflow velocity in this study was at the low end of practically relevant conditions. Higher crossflow velocities would lead to lower extents of membrane fouling. Therefore, we conclude that the effects of pre-heating and calcium addition would be attenuated compared with the results obtained in this study, with a more pronounced deposit formation.

Furthermore, to extend the possibilities of a pre-heating prior to microfiltration, additional studies are suggested concerning diffusion kinetics as a function of heating temperature and tempering time, as well as cooling time, to determine which heating conditions and which time profile can be best used to decrease the level of soluble caseins prior to microfiltration. Furthermore, insights would be gained by determining the necessary time required until an equilibrium between serum and micellar casein is reached if the skim milk is cool after the pre-heating step.

Additional analytical methods would also be desirable to differentiate between added calcium and casein monomer aggregation, whey protein aggregation, and the integration of casein monomers within casein micelles.

## Figures and Tables

**Table 1:** Protein distribution in skim milk used during the experiments.

	Total Casein	κ-Casein	α_S1_-Casein	α_S2_-Casein	β-Casein	Total Whey Protein	α-La, BSA, and LF	β-Lg A and β-Lg B
Concentration [g L^−1^]	33.7	4.2	12.6	4.3	12.4	5.6	1.2	4.4

**Table 2:** Flux, milk protein permeation, and concentration in the permeate during microfiltration (MF) at 10 °C, without and with pre-heating prior to microfiltration. Measured after a process time of 50 min under stable filtration conditions.

		Without Pre-Heating	With Pre-Heating
Flux	[L m^−2^ h^−1^]	8.1 ± 0.2	8.1 ± 0.1
Casein permeation	[%]	1.6 ± 0.1	1.2 ± 0.1
Casein conc. in the permeate	[g L^−1^]	0.55 ± 0.03	0.35 ± 0.04
β-casein conc.	[g L^−1^]	0.32 ± 0.01	0.19 ±0.02
α_S1_-casein conc.	[g L^−1^]	0.07 ± 0.00	0.03 ± 0.02
α_S2_-casein conc.	[g L^−1^]	0.06 ± 0.05	0.07 ± 0.00
κ-casein conc.	[g L^−1^]	0.1 ± 0.02	0.06 ± 0.01
Whey protein permeation	[%]	24.0 ± 0.1	22.1 ± 1.4
Whey protein conc. in the permeate	[g L^−1^]	1.4 ± 0.00	1.17 ± 0.05

**Table 3:** Whey protein/casein (WP/C) ratio [-] in the permeate after filtration, with and without pre-heating, at various concentrations of added calcium. A significant difference at a level of *p* < 0.05 between the WP/C ratio with and without pre-heating was determined.

Added Calcium Concentration	0 mM	5 mM	10 mM
WP/C ratio without pre-heating	2.6 ± 0.1	2.0 ± 0.2	1.9 ± 0.1
WP/C ratio with pre-heating	3.3 ±0.5	2.9 ± 0.6	3.0 ± 0. 1
